# General health status and psychological impact of COVID19 pandemic and curfew on children aging 3 to 12 years

**DOI:** 10.3389/frcha.2022.1034492

**Published:** 2022-12-12

**Authors:** Abdullah Shamsah, Maryam Aburezq, Zahraa Abdullah, Fatemeh Khamissi, Batool Almaateeq, Fatima AlAlban, Sarah Almashmoom, Ali Ziyab

**Affiliations:** ^1^ Pediatrics Emergency Unit, Mubarak Al Kabeer Hospital, Jabryia, Kuwait; ^2^ Department of Pediatrics, Farwanyia Hospital, Sabah Al nasser, Kuwait; ^3^ Department of Pediatrics, Amiri Hospital, Asimah, Kuwait; ^4^ Faculty of Medicine, Health Science Center, Kuwait University, Jabryia, Kuwait; ^5^ Department of Community Medicine and Behavioral Sciences, Faculty of Medicine, Health Science Center, Kuwait University, Jabryia, Kuwait

**Keywords:** COVID-19, pandemic, psychological impact, pediatrics, coping, mental health

## Abstract

**Background:**

COVID-19 is an infectious disease that was declared as a pandemic and public health emergency in late 2019 and has impacted children's mental health worldwide. This study aimed to assess the general and mental health status of children during different stages of COVID-19 pandemic, and to identify the associated factors.

**Methods:**

A cross-sectional study conducted on children aging 3 to 12 years in Kuwait during three different stages of COVID19 pandemic (pre-total curfew, during total curfew, and post-total curfew). The psychological status was assessed using the fifth edition of the Diagnostic and Statistical Manual of Mental Disorders criteria.

**Results:**

Of 2157 children between the age of 3 to 12 years old, 853 (39.5%) reported increased level of aggression, 789 (36.6%) over-crying, 749 (34.7%) sadness, 493 (22.9%) anxiety, 429 (19.9%) anhedonia, 383 (17.8%) confusion, 274 (12.7%) nightmares, 177 (8.2%) avoidance, 174 (8.1%) physical symptoms and 121 (5.6%) bedwetting during the pandemic. General health status was also affected with reported disturbed sleeping pattern (84.6%), altered appetite (50.9%) and weight changes (36.9%), mainly weight gain. Risk factors included being non-national, as well as having lower parental educational level and lower socioeconomic status; while protective factors involved meeting classmates, indoor and outdoor activities, and less screen time.

**Conclusions:**

COVID19 crisis had drastic impact on children's mental and general health, requiring serious action regarding evaluating this generation and intervening accordingly.

## Introduction

Coronavirus disease 2019 (COVID-19) outbreak started in Wuhan, China in 2019, and rapidly grew into a global pandemic in March 2020 (1). It is caused by a novel strain of severe acute respiratory syndrome coronavirus 2 (SARS-CoV-2), which caused the previous epidemics of severe acute respiratory syndrome (SARS) in 2002–2004 and Middle East Respiratory Syndrome (MERS) in 2012 (2). Evidence showed that SARS-CoV spreads primary by human-to-human transmission *via* droplets or direct contact with infected individuals (3); therefore, enhanced preventative measures were implemented worldwide in an attempt to prevent and contain the spread of the infection. As a result, this pandemic has impacted individuals in different aspects, including physical health, social and economic status, along with its alarmingly increasing psychological impact among general population (Hossain et al., 2020). The latest was documented in previous research literatures, as exposure to natural disasters can pose mental health challenges and negative outcomes especially in children (4). Anxiety disorders in children were consistently correlated with the exposure of such extensive natural disasters, along with the emotional distress, physical and psychological sequelae (5). A previous multilevel meta-analysis reported psychiatric morbidities, post-traumatic stress disorder, and depression in several impacted populations during different health related crises including SARS, MERS and COVID19 (6). Variable levels of anxiety and depressive symptoms were also detected in a systemic review during COVID19 pandemic among children and adolescent, either due to their fear of the illness or to social isolation (7). In China, children and adolescents have reported significant increase in separation anxiety, physical injury, fear, social phobia, panic disorder, and generalized anxiety during COVID19 pandemic (8). Alarming results of increased suicidal ideation and suicidal attempts were also reported in another study that was conducted in Rural China among high school students during this pandemic (9).

Regarding the reported risk factors, a recent systemic review conducted during COVID19 pandemic revealed that the increase of anxiety, depression, fatigue and distress among children and college students were reported more in those with low socioeconomic status and those who have a healthcare worker relative or friend (10). Additionally, another rapid systemic review showed that although COVID19-related school closure has reduced hospital admissions and emergency department visits; it has significantly increased levels of anxiety, loneliness, frustration, stress and sadness among children; in addition to the decrease in their activity level and increase in obesity prevenances and body mass index (BMI) (11). With respect to the implemented quarantine, a study conducted in Italy and Spain reported worsened level of concentration, irritability, boredom, restlessness, loneliness, nervousness, uneasiness, worries, anxiety, and anger in children and adolescent aging 3 to 18 years during the quarantine (12). Likewise, a study conducted in India revealed that quarantined children and adolescents experienced increased worrying, helplessness, and fear, as well as higher psychological distress level compared to the non-quarantined individuals (13).

Similar findings were reported in surrounding areas, including the Arabian Gulf countries, where a recent study conducted in Qatar reported negative impact of the pandemic on the lifestyle of children aging 5 to 12 years, including decreased level of activity, increase screen time, increase in number of meals per day and disturbed sleeping pattern during the quarantine (14). Another cross-sectional study also conducted in Qatar showed higher levels of anxiety among children during the pandemic, especially in non-nationals and those quarantined in institutional centers (15). Furthermore, a nationwide cross-sectional study in United Arab of Emirate reported parental anxiety to be a risk factor of children anxiety during COVID19 pandemic (16).

COVID-19 is the first pandemic for which Kuwait imposed a mandatory quarantine and implemented a curfew. With all the previously reported negative mental health outcomes of this pandemic on children worldwide, it is very important to study the mental health status and the resultant symptoms of this pandemic on children in Kuwait as well. Therefore, the aim of our study is to ascertain the behavioral and psychological effect of COVID19 pandemic on children in Kuwait and provide an insight of their general and mental health status during and after implementing the curfew; in addition to identifying the associated protective and risk factors. This study is one of few studies conducted in the country and the surrounding regions focusing on the psychological aspect of the pandemic, especially on children. Thus, our findings can incite and direct public health practitioners, planners and policymakers to consider further evaluation and proper rehabilitation for vulnerable groups, implement new strategies to deal with future events, and to educate the parents along with their children how to accommodate in such stressful situations. Timely action and intervention are crucial to lessen the consequences of this pandemic and protect future generations.

## Material and methods

### Study design, study population and data collection

A cross-sectional study conducted on children aging 3 to 12 years from April to June 2020 in Kuwait during different stages of COVID19 pandemic (pre-total curfew, during total curfew and post-total curfew). A web-based survey was professionally disseminated with clear guidelines and instructions using social media platforms, including Twitter, Instagram, and WhatsApp to enroll parents living in Kuwait. Snowball sampling technique, a non-probability sampling method, was used to recruit participants. This method depends on existing participants to recruit further subjects among their acquaintances; hence, resembling a rolling snowball that picks participants as it spreads across the population. This methodology yields a convenience sample. A total of 2,157 complete responses were included, accounting for 51% of the total collected responses (4,220), while the remaining responses of the participants who quitted the survey before reaching the last page were excluded from the study. Caregivers were instructed to enroll only one child from each family, the eldest child below the age of 13 years, to avoid biased results towards the most affected children, and their consents were obtained before participation. While conducting the study all over Kuwait, caregivers were advised to contact a virtual campaign held by the authors of this study along with a group of pediatricians, specialists and medical students in Kuwait during the pandemic that aimed to relieve the children, teach them how to cope with this new stressful situation and guide the caregivers when and how to seek medical advice regarding the addressed symptoms.

### Ethical approval

The study was approved by the Standing Committee for Coordination of Health and Medical Research in Kuwait. Moreover, the approval of the Assistant Undersecretary for Planning and Quality Affairs, the Assistant Undersecretary for Technical Affairs, the Assistant Undersecretary for Public Health Affairs, the Director of National Center of Health Information, the Chairman of the Board of Pediatrics and Premature Pediatrics Departments, and the Director of Information Systems Department was obtained. The aim of the study along with the total number of questions and the expected time to solve the survey were all demonstrated and provided in the consent forms, which assured voluntary participation and confidentiality of the collected data; in addition to introductory paragraphs that were added before each group of questions explaining the aim of each section.

### Study instrument

The data were collected using an open web-based survey, due to the implemented restrictions of face-to-face contact during the pandemic. The survey was designed using SurveyMonkey, which is a web-base, flexible, scalable and secure survey developmental tool that is commonly used for collecting research data (17). The questionnaire was developed originally in English then translated into Arabic. Two independent bilingual individuals, who were not involved in the study, translated the Arabic version back to English and helped in pretesting and piloting the final versions of the study. The study questions were evaluated, reviewed and approved by an external pediatrics psychiatrist, and they were pretested by six investigators before starting data collection.

This survey involved a total of 61 questions, including the subtitled questions, that were divided into five sections covering (1) sociodemographic characteristics, (2) general health status during the pandemic, including altered appetite, weight and sleeping pattern, (3) psychological status, including important symptoms of anxiety and depression, (4) free times activities including indoor and outdoor activities as well as usage of electronic devices, (5) and data obtained from children above 7 years addressing their thoughts, beliefs, and any homicidal or suicidal ideation.

The third section was assessed *via* 5-point Likert scale questions using Diagnostic and Statistical Manual of Mental Disorders, Fifth Edition (DSM-5), a standard classification of mental disorders that is approved by the American Psychiatric Association (APA) and the World Health Organization (WHO) (18), to evaluate the psychological status rather than establishing diagnosis. These variables included: sadness/low mood, anhedonia (loss of interest in previously enjoyed activities), over-crying/demanding, anxiety/worrying, aggression, social withdrawal/avoidance, confusion/low concentration, nightmares, bedwetting and physical symptoms, including abdominal pain/vomiting; as recurrence of such somatic complaints in the absence of medical condition is included in the criteria of several pediatric anxiety disorders (19).

Most of the study questions were labeled as mandatory, for which the participants were notified in case any of these mandatory questions were left blank before proceeding to the next section. The collected data were IP address coded; they were exported to Excel sheet, cleaned, and then exported to SPSS for further analysis.

### Statistical analysis

Data were analyzed using IBM® SPSS Statistics for Windows, Version 25.0 (IBM Corp., Armonk, NY, USA). The statistical significance level of *α* = 0.05 was used for all association analyses. Descriptive analyses were conducted to calculate frequencies and proportions of categorical variables (i.e., psychological and general health status) in the total sample and after stratification by the stage of pandemic, frequency of interacting with classmates during the pandemic, and monthly family income. Chi-square (*χ*^2^) tests were used to assess whether proportions of psychological and general health status variables differed across categories of demographic variables.

## Results

Sociodemographic characteristics of participants are illustrated in Table 1. A total of 2,157 nonsibling children participated in this study, 1,129 (52.3%) of them were boys and 1,028 (47.7%) girls. Two-thirds the population were between the age of 3 to 7 years (1,312, 62.8%), while 777 (37.2%) were 8 to 12 years. Children living in extended-family houses accounted for 43.2% of the population (931/2,157); while 455 participants (21.1%) were living in single-family houses and 771 (35.7%) in apartments. Most children had parents with a university degree or higher (1,318, 61.1% and 1646, 76.3% for paternal and maternal degrees, respectively), and 898 participants (41.6%) had a monthly family income of 2,000 Kuwaiti Dinar (KWD) or above.

**Table 1:** Sociodemographic factors of participants in the total study sample and according to curfew stages stratification.

Variables	Total Study Sample (*n* = 2157)	Pre-total curfew (*n* = 831)	During total curfew (*n* = 655)	Post-total curfew (*n* = 671)
	% (*n*)	% (*n*)	% (*n*)	% (*n*)
Child's age (years)				
3–7	62.8 (1312)	67.4 (546)	58.5 (369)	61.3 (397)
8–12	37.2 (777)	32.6 (264)	41.5 (262)	38.7 (251)
Missing (*n*)	68	21	24	23
Child's gender				
Male	52.3 (1129)	50.9 (423)	53.1 (348)	53.4 (358)
Female	47.7 (1028)	49.1 (408)	46.9 (307)	46.6 (313)
Nationality				
Kuwaiti	89 (1919)	90.1 (749)	87 (570)	89.4 (600)
Non-Kuwaiti	11 (238)	9.9 (82)	12.9 (85)	10.5 (71)
Governorate				
Asimah (Capital)	26.7 (575)	28 (233)	30.2 (198)	21.5 (144)
Farwanyia	9.6 (208)	6.7 (56)	11.8 (77)	11.2 (75)
Hawali	34.2 (737)	37.4 (311)	32.1 (210)	32.2 (216)
Mubarak Al-Kabeer	17.1 (368)	17.1 (142)	14.4 (94)	10.7 (132)
Al Ahmadi	9.2 (198)	9 (75)	7.8 (51)	10.7 (72)
Al Jahraa	3.3 (71)	1.7 (14)	3.8 (25)	4.8 (32)
Housing				
Single family house	21.1 (455)	22.7 (189)	20.9 (137)	19.2 (129)
Extended family house	43.2 (931)	43 (357)	44.7 (293)	41.9 (281)
Apartment or other	35.7 (771)	34.3 (285)	34.3 (225)	38.9 (261)
Family monthly income (in KWD)				
<1,000	14.4 (310)	13.5 (112)	13.4 (88)	16.4 (110)
1,000–1,499	23.5 (506)	24.3 (202)	20.8 (136)	25 (168)
1,500–2,000	20.5 (443)	18.7 (155)	21.8 (143)	21.6 (145)
>2,000	41.6 (898)	43.6 (362)	44 (288)	37 (248)
Educational level of child's father				
High school and below	15.5 (334)	14.5 (120)	22.1 (92)	18.4 (122)
Diploma	22.5 (484)	22.8 (189)	19.6 (128)	25.2 (167)
University (bachelor’s degree)	61.6 (1,329)	62.7 (520)	66.4 (434)	56.5 (375)
Missing (*n*)	10	2	1	7
Educational level of child's mother				
High school and below	5.5 (120)	4.7 (39)	6 (39)	6.2 (42)
Diploma	17.8 (385)	18.4 (152)	16.3 (107)	18.9 (126)
University (bachelor’s degree)	76.3 (1,645)	76.9 (637)	77.7 (509)	74.8 (499)
Missing (*n*)	7	3	0	4
Marital status of the parents				
Married	91.1 (1,966)	91.7 (762)	90.7 (594)	90.9 (610)
Divorced or Widowed	8.8 (191)	8.3 (69)	9.3 (61)	9 (61)

Regarding general health status, 84.6% of participants (1,825/2,157) reported having disturbed sleep pattern, 50.9% (1,098/2,157) altered appetite and 36.9% (796/2,157) had weight changes since the pandemic started; 62.6% of whom (498/796) gained weight.

**Figure 1:**
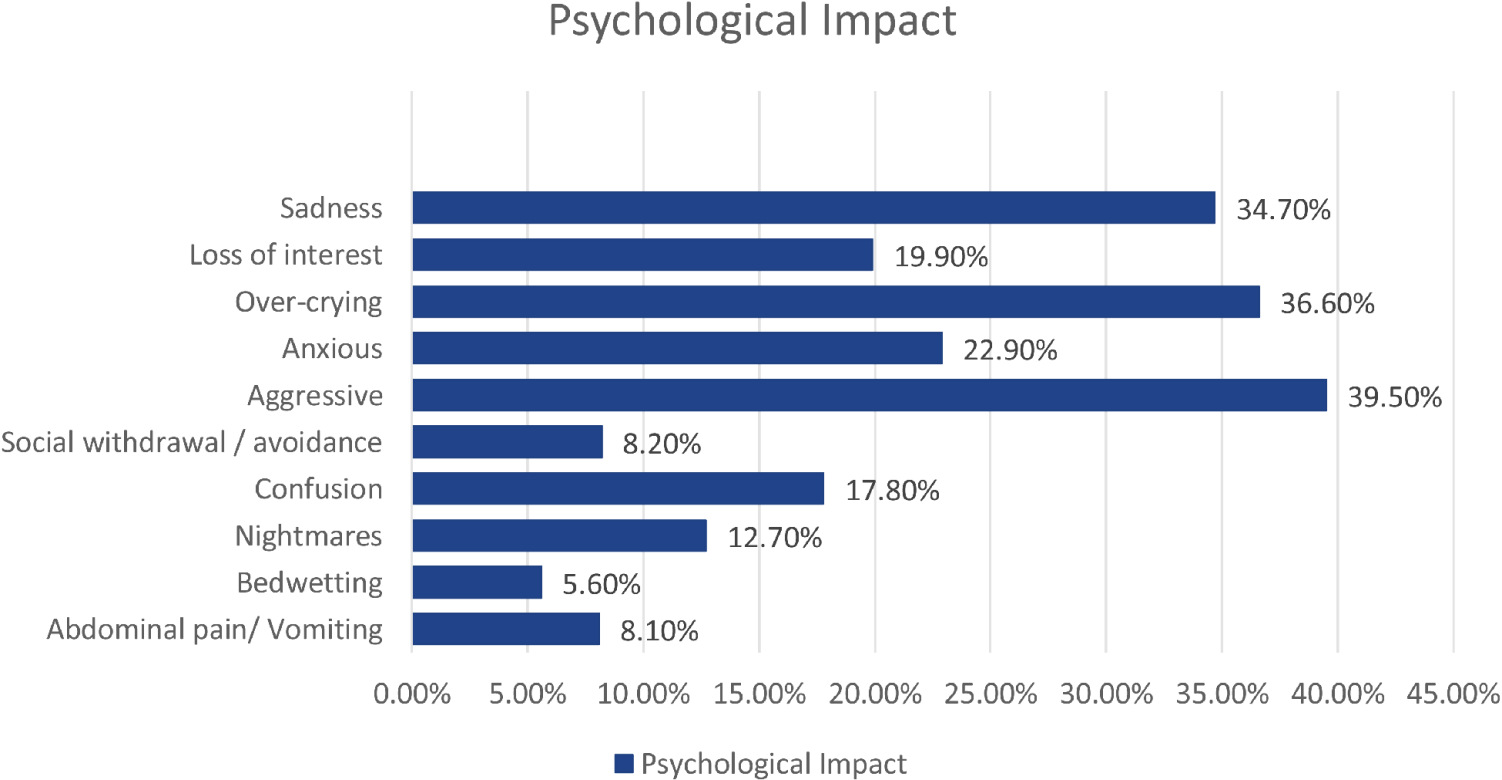
Frequencies of variables assessing the psychological status of children during COVID-19 pandemic in Kuwait (*n *= 2157).

Figure 1 illustrates the psychological symptoms experienced children during COVID-19 pandemic in Kuwait; 39.5% of participants (853/2,157) were more aggressive, 36.6% (789/2,157) over-crying, 34.7% (749/2,157) sad, 22.9% (493/2,157) anxious, 19.9% (429/2,157) had anhedonia, and 17.8% (383/2,157) had decreased concentration and confusion, while social withdrawal/avoidance was recognized in 8.2% (177/2,157) and physical symptoms in 8.1% (174/2,157) of them. Moreover, some caregivers reported frequent nightmares and newly-onset bedwetting behavior among their children (274/2,157, 12.7% and 121/2,157, 5.6%, respectively).

Table 2 demonstrates the association between the psychological symptoms with pandemic duration and curfew status. Anxiety and physical symptoms were significantly increasing throughout the pandemic (20.1% vs. 21.8% vs. 27.3%, *p* = 0.022 and 6.3% vs. 9% vs. 9.4%, *p* = 0.031, respectively). Nightmares (11.4% vs. 11.3% vs. 15.6%, *p* = 0.004) and anhedonia (19% vs. 17.6% vs. 23.2%, *p* = 0.051) were significantly higher in post-total curfew, while over-crying at the beginning of the pandemic (39.8% vs. 32.1% vs. 37%, *p* = 0.044). On homicidal and suicidal ideation assessment of children aging 8 and 12 years, 77 out of 657 participants had homicidal behaviors, while 25 children (3.8%) had suicidal ideation.

**Table 2:** Psychological status during COVID19 pandemic with regards to the curfew status in Kuwait (*n* = 2157).

Variables	Curfew/Lock Down Status			*P*-value^a^
	Pre-total curfew (Partial Curfew) (*n* = 831)	Total Curfew (*n* = 655)	Post-total Curfew (Partial Curfew) (*n* = 671)	
	% (*n*)	% (*n*)	% (*n*)	
Sadness	35.1 (292)	33.4 (219)	35.5 (238)	0.578
Loss of interest/anhedonia	19 (158)	17.6 (115)	23.2 (156)	0.051
Over-crying	39.8 (331)	32.1 (210)	37 (248)	0.044
Anxious	20.1 (167)	21.8 (143)	27.3 (183)	0.022
Aggressive	38 (316)	39.8 (261)	41.1 (276)	0.505
Social withdrawal/avoidance	7.2 (60)	8.4 (55)	9.2 (62)	0.625
Confusion	17.7 (147)	16.3 (107)	19.2 (129)	0.227
Nightmares	11.4 (95)	11.3 (74)	15.6 (105)	0.004
Bedwetting	5.2 (43)	6.1 (40)	5.5 (37)	0.402
Abdominal pain/Vomiting	6.3 (52)	9 (59)	9.4 (63)	0.031

^a^ Calculated using chi-square tests.

Children between 3- to 7-year-old, compared to those aging 8 to 12 years, reported higher levels of sadness (36.2% vs. 31.9%, *p* = 0.034), over-crying (44.1% vs. 24.7%, *p* < 0.001), aggression (41.2% vs. 37.3%, *p* < 0.001), bedwetting (7.2% vs. 3%, *p* < 0.001) and nightmares (15% vs. 9.1%, *p* < 0.001). However. older children reported more social withdrawal (12.5% vs. 5.3%, *p* < 0.001), confusion (21% vs. 15.8%, *p* = 0.011) and physical symptoms (10.6% vs. 6.6%, *p* = 0.009) compared to younger ones. Moreover, boys reported more aggression (43.1%% vs. 35.6%, *p* = 0.001), confusion (19.6% vs. 15.8%, *p* = 0.01) and bedwetting (7.2% vs. 3.8%, *p* < 0.001) than girls, with a trend of more social withdrawal among boys compared to girls as well despite the absence of statistical significance (8.5% vs. 7.9%, *p* = 0.080).

Regarding living status, sadness (40.9% vs. 30.8% vs. 31.6%), anhedonia (22.7% vs. 18.7% vs. 18.2%), over-crying (41.8% vs. 31.6% vs. 34.7%), anxiety (27.6% vs. 22.6% vs. 19%), aggression (46.8% vs. 33% vs. 36.7%) and confusion (22.2% vs. 16.9% vs. 14.5%) were relatively higher among children living in apartments compared to those in single-family and extended-family houses (*p* < 0.001, 0.008, 0.002, < 0.001, < 0.001 and <0.001, respectively). All studied psychological symptoms, except for bedwetting, were associated with low family income (below 1,000 KWD), (*P* < 0.001 for each of sadness, anhedonia, over-crying, anxiety, confusion, nightmares and physical symptoms, *p* = 0.024 for avoidance and 0.634 for bedwetting; Table 3).

**Table 3:** Psychological status of children during the pandemic with regards to the monthly family income (*n* = 2157).

Variables	Monthly Family Income				*P*-value^a^
	<1,000 KD (*n* = 310)	1,000–1499 KD (*n* = 506)	1,500–2000 KD (*n* = 443)	>2,000 KD (*n* = 898)	
	% (*n*)	% (*n*)	% (*n*)	% (*n*)	
Sadness	42.3 (131)	37.7 (191)	31.6 (140)	32 (287)	<0.001
Loss of interest/anhedonia	27.1 (84)	19.8 (100)	20.1 (89)	17.4 (156)	<0.001
Over-crying	45.8 (142)	37.5 (190)	33.6 (149)	34.3 (308)	<0.001
Anxious	33.5 (104)	26.1 (132)	20.3 (90)	18.6 (167)	<0.001
Aggressive	53.5 (166)	42.1 (213)	35.4 (157)	35.3 (317)	<0.001
Social withdrawal/avoidance	10.6 (33)	8.9 (45)	6.8 (30)	7.7 (69)	0.024
Confusion	27.7 (86)	17.4 (88)	15.3 (68)	15.7 (141)	<0.001
Nightmares	19.4 (60)	13.4 (68)	11.1 (49)	10.8 (97)	<0.001
Bedwetting	6.8 (21)	5.9 (30)	6.1 (27)	4.7 (42)	0.634
Abdominal pain/ Vomiting	13.9 (43)	7.7 (39)	5 (22)	7.8 (70)	<0.001

^a^ Calculated using chi-square tests.

With respect to parental educational level (high-school or lower vs. diploma vs. university of higher), an inverse association was noted with aggression (paternal 47.3% vs. 38.6% vs. 37.9%, *p* = 0.030 and maternal 46.7% vs. 44.4% vs. 38%, *p* = 0.026). Social withdrawal was significantly higher among those with parental high-school degree or below (parental 9.9% vs. 7% vs. 8.1%, *p *=< 0.001 and maternal 9.2% vs. 7.8% vs. 8.3%, *p* < 0.001). Sadness was remarkably higher among those with lower maternal educational level (43.3% v. 35.1% vs. 34.1%, *p* = 0.023) with no association with paternal level (36.8% vs. 33.3% vs. 34.8%, *p* = 0.823). On the other hand, anhedonia, nightmares and physical symptoms were associated with lower paternal educational level (24% vs. 20.5% vs. 18.7%, *p* = 0.002; 16.5% vs. 12.2% vs. 12%, *p* = 0.035; and 11.1% vs. 9.5% vs. 6.8%, *p* = 0.041 respectively), in addition to confusion, which was particularly higher among those with paternal degree of high school or lower (22.8% vs. 15.3% vs. 17.3%, *p *=< 0.001).

Children with altered sleeping pattern reported higher sadness levels than those with constant pattern (86.8% vs. 13.2%, *p* = 0.008). With respect to physical activity (PA), spending one to three hours daily on indoor activities was inversely associated with sadness (*p* = 0.009) and social withdrawal/avoidance (*p* < 0.001), and spending more than three hours on them was associated with less anxiety and confusion levels (*p* = 0.005 and *p* < 0.001, respectively). In the same fashion, spending one to three hours daily on outdoor activities was associated with less confusion (*p* = 0.005) and social withdrawal/avoidance (*p* = 0.011), while spending more than 3 h on them was inversely correlated to anhedonia and anxiety (*p* = 0.017 and *p* = 0.012, respectively).

Meeting classmates during the pandemic was also inversely associated with sadness (*p* = 0.047), over-crying (*p* < 0.001), aggression (*p* < 0.001), confusion (*p* < 0.001) and nightmares (*p* = 0.008), while not meeting them was directly associated with anxiety, anhedonia, avoidance and bedwetting (*p* = 0.003, *p* = 0.003, *p* = 0.04, and *p* = 0.024, respectively; Table 4). Moreover, significant association was found between the reported symptoms and spending more than three hours daily on electronic devices, showing higher levels of sadness (*p* = 0.002), anhedonia (*p* < 0.001), anxiety (*p* < 0.001), aggression (*p* < 0.001), social withdrawal (*p* < 0.001) and confusion (*p* = 0.011), with an increased trend of over-crying as well (*p* = 0.055).

**Table 4:** Psychological status with regards to the frequency of interacting with classmates (*n* = 2057) (missing 100).

Variables	Frequency of Interacting with Classmates					*P*-value^a^
	Never (*n* = 450)	Rarely (*n* = 459)	Sometimes (*n* = 827)	Often (*n* = 291)	Always (*n* = 130)	
	% (*n*)	% (*n*)	% (*n*)	% (*n*)	% (*n*)	
Sadness	40 (180)	36.4 (167)	33.3 (275)	30.6 (89)	29.2 (38)	0.047
Loss of interest/anhedonia	24 (108)	20.7 (95)	19 (157)	16.2 (47)	16.9 (22)	0.003
Over-crying	47.1 (212)	37.7 (173)	34.1 (282)	32 (93)	22.3 (29)	<0.001
Anxious	27.8 (125)	22.7 (104)	22.3 (184)	18.6 (54)	20 (26)	0.003
Aggressive	50.4 (227)	40.7 (187)	36.2 (299)	35.1 (102)	29.2 (38)	<0.001
Social withdrawal/avoidance	10.7 (48)	7.6 (35)	7.4 (61)	8.9 (26)	5.4 (7)	0.041
Confusion	22.9 (103)	17.7 (81)	16.4 (136)	15.1 (44)	14.6 (19)	<0.001
Nightmares	17.3 (78)	12.4 (57)	11.9 (98)	10.7 (31)	7.7 (10)	0.008
Bedwetting	6.7 (30)	5 (23)	6.2 (51)	4.5 (13)	2.3 (3)	0.024
Abdominal pain/Vomiting	7.6 (34)	7.8 (36)	8.4 (70)	7.2 (21)	10 (13)	0.854

^a^ Calculated using chi-square tests.

## Discussion

### Principal findings

Drastic changes have been reported during COVID19 pandemic regarding children's general health status, including disturbed sleeping pattern, altered appetite and weight changes, mostly weight gain. Regarding the psychological aspect, negative symptoms were reported during the pandemic, including sadness, anhedonia, over-crying, anxiety, aggression, social withdrawal, confusion, nightmares, bedwetting and physical symptoms, with significant different trends with regards to the pandemic duration and curfew status. Both genders were affected during this pandemic, however aggression, confusion, and bedwetting were remarkably higher among boys. Regarding age groups, younger children reported higher levels of sadness, over-crying, aggression, bedwetting, and nightmares, while older children reported more social withdrawal, confusion and physical symptoms. Negative symptoms were significantly more reported among those with lower socioeconomic status. When shedding the light on homicidally and suicidality among those above 7-year-old, 77 children had homicidal behaviors and 25 had suicidal ideation during pandemic. Spending time on indoor and outdoor activities and meeting classmates were found to be protective factors against the reported symptoms, while having an altered sleeping pattern and prolonged screen time were reported as negative factors.

### Duration and curfew status

A systematic review and meta-analysis of the psychological and behavioral impact on children showed that 34.5% were anxious, 41.7% depressed, 42.3% irritable and 30.8% experienced inattention as a consequence of the lockdown and quarantine (20). In the same fashion, our study revealed that anhedonia and nightmares were remarkably higher in post-total curfew stage, while over-crying was higher at the beginning of the pandemic. Moreover, anxiety and physical symptoms were significantly increasing throughout the pandemic. This could be due to the newly enforced isolation and unusual situations with the pandemic uncertainty.

### Age and gender

Younger participants in this study revealed higher levels of sadness, over-crying, aggression, bedwetting and nightmares compared to older ones, while the latter group was significantly higher in social withdrawal, confusion and physical symptoms. In contrast, three different studies reported that adolescents and older children exhibited more depressive symptoms than younger ones during this pandemic (21). Another study conducted by Imran et al., (2020) reported more anxiety, anger and restlessness among older children compared to younger ones, but more social withdrawal among younger children; the latter is in accordance with our findings. Two studies conducted by Wang, Pan, Wan, Tan, Xu, Ho, et al. (2020) (22) and Wang, Pan, Wan, Tan, Xu, McIntyre, et al. (2020) (23) reported higher anxiety levels in younger children compared to older ones. Another study also revealed a higher trend of negative psychological symptoms among individuals aging 14 to 20 years compared to older age group (24). Boys involved in this study reported higher levels of aggression, confusion and bedwetting than girls, with a trend of more social withdrawal among boys as well. Two different studies included pediatric population found that girls were more likely to develop anxiety and depressive symptoms compared to boys (25, 26), while another study conducted by Ahmed et al. (2020) (27) showed no significant gender difference in mood and anxiety. These findings support the fact that boys and girls of all ages are prone to develop psychological issues in such public health events.

### Environmental impact

Participants living in apartments reported more psychological symptoms than those living in houses. Our finding is consistent with a European study conducted during this pandemic, showing that living with more people at one house and having a garden/terrace reduced the impact of curfew on children's well-being, development, and mental health, except for mood alteration that was not affected (28). Moreover, our study manifested a significant impact on children with low family income, reporting more sadness, anhedonia, over-crying, anxiety, confusion, nightmares, physical symptoms and social withdrawal. A similar finding explained in another study to be due to their reliance on school's healthy meals, mental health support and playgrounds for physical exercise (21), Furthermore, higher parental educational level reflected less psychological and behavioral changes in children in our study, which agreed with previous studies were higher parental degrees were inversely associated with adversely affected mental health (29, 30). Such associations emphasize on the huge impact of socioeconomic status on children's mental health and behaviors under stressful events.

### General health status

Most of our participants reported an altered sleeping pattern. A study initiated by Kaditis et al. (2021) (31) demonstrated that children with altered sleep-wake pattern had increased emotional symptoms and difficulty in self-regulation, which goes in line with the significant increase in sadness level among our participants with altered sleeping pattern. Almost half of our sample reported altered appetite and some others had weight changes, mostly weight gain, which can be explained by the unhealthy habits and decreased activity level during the pandemic; as an Italian study reported a significant increase in participants consumption of potato chip, red meat, and sugary drinks during the lockdown (32).

### School closure

Regardless the effectiveness of school closure on controlling influenza virus outbreaks, its effect on SARS, MERS and COVID19 remains unclear (33, 34). Recent modelling studies predicted that other physical distancing interventions were more effective than school closures, as the latter prevented only 2%–4% of deaths (33). School closure has a potential effect on children's nutrition, BMI, PA and daily routine (21, 35); especially on those with mental health diseases and special needs as going to school is an important relapse prevention measure for them (36). Our study also considered meeting classmates during the pandemic as a protective factor as it revealed less sadness, aggression, crying, confusion and nightmares; while children who never met their classmates showed significant higher levels of anxiety, anhedonia and bedwetting. Thus, school closure decision in such situations should be implemented after benefit-risk assessment with efficient use of social media to maintain social networks and lessen the burden of social distancing, and to consider early reopening with restrictive physical distancing to maintain infection control.

### Free times activities

Although a prospective cohort study conducted among adolescents found no strong association between PA and mental health symptoms, it showed a protective correlation with emotional problems subscale of the Strengths and Difficulties Questionnaire (SDQ), including symptoms of depression and anxiety (32). Another study conducted by Chen et al. (2020) also reported less depression and anxiety among regular exercisers. These findings were in line with our results where indoor and outdoor activities were found to be protective factors. On the other hand, prolonged screen time has affected our participants' psychological status adversely. Similarly, another study has revealed an association between prolonged screen time and increased psychosocial consequences especially among younger children (37). In Saudi Arabia, spending long time on gaming activities during the quarantine has affected children, where 20.4% became introverted and 14.5% more aggressive ((38). In the same fashion, an association between smartphone and internet addiction with depression was reported in China (8). These findings reveal the seriousness of screen time's effect on children; thus, such sequel must be tackled down by mental health professionals.

### Suicidal ideation

Suicidal tendency was significantly and constantly raising during this pandemic among school children worldwide, including Kerala, where 173 children died from suicide (39). Twenty-five children enrolled in our study reported getting suicidal thoughts during the pandemic, which is concerning.

### Limitations

• The snapshot nature of the cross-sectional study lacks the assessment of the changes over time; thus, further cohort studies are required to address the causality relationship and to assess the long-term outcomes of this pandemic.
• The subjective understanding of the questions due to lack of face-to-face interaction while distributing the online questionnaire; thus, an explanatory note was added before each section.
• The length of our questionnaire led to having around 49% of incomplete responses; thus, the studied findings were limited on the completed ones only, to include 2,157 complete responses out of 4,227.

## Conclusion

COVID19 pandemic is a public health emergency that led to serious psychological and general health consequences. This study highlighted the drastic changes in children's general and mental health status during this pandemic, reflecting the importance of early evaluation, thus providing appropriate intervention and mental care to prevent further progression and negative future outcomes. Children are encouraged to have constant sleep pattern, utilize their free time effectively on physical and educational activities and to avoid prolonged screen time. Caregivers are also advised to seek early professional help before such a stress leads to further functional and developmental impairment. Moreover, educational authorities should evaluate the benefit-risk effect of school closure in such situations; if closure is to be considered, web-based portals are encouraged to deliver online classes temporarily ensuring education continuity and minimizing its adverse impact on children. Further investigations and research evaluating long-term effect of COVID19 pandemic is required, as timely action and intervention can lessen the consequences, improve the outcome and protect our future generation.

## Data Availability

The raw data supporting the conclusions of this article will be made available by the authors, without undue reservation.
